# The chromosomes and the mitogenome of *Ceratitis fasciventris* (Diptera: Tephritidae): two genetic approaches towards the *Ceratitis* FAR species complex resolution

**DOI:** 10.1038/s41598-017-05132-3

**Published:** 2017-07-07

**Authors:** Elena Drosopoulou, Christina Pantelidou, Angeliki Gariou-Papalexiou, Antonios A. Augustinos, Tatiana Chartomatsidou, Georgios A. Kyritsis, Kostas Bourtzis, Penelope Mavragani-Tsipidou, Antigone Zacharopoulou

**Affiliations:** 1 Department of Genetics, Development and Molecular Biology, School of Biology, Faculty of Sciences, Aristotle University of Thessaloniki, Thessaloniki, Greece; 2 Department of Biology, University of Patras, Patras, Greece; 3 Insect Pest Control Laboratory, Joint FAO/IAEA Division of Nuclear Techniques in Food and Agriculture, Seibersdorf, Vienna, Austria

## Abstract

*Ceratitis fasciventris* is a serious agricultural pest of the Tephritidae family that belongs to the African *Ceratitis* FAR species complex. Species limits within the FAR complex are obscure and multidisciplinary approaches have attempted to resolve phylogenetic relationships among its members. These studies support the existence of at least three additional species in the complex, *C*. *anonnae*, *C*. *rosa* and *C*. *quilicii*, while they indicate the presence of two structured populations (F1 and F2) within the *C*. *fasciventris* species. In the present study we present the mitotic karyotype, polytene chromosome maps, *in situ* hybridization data and the complete mitochondrial genome sequence of an F2 population of *C*. *fasciventris*. This is the first polytene chromosome map and complete mitogenome of a member of the FAR complex and only the second reported for the *Ceratitis* genus. Both polytene chromosomes and mitochondrial sequence could provide valuable information and be used as reference for comparative analysis among the members of the complex towards the clarification of their phylogenetic relationships.

## Introduction

The Tephritidae family consists of more than 500 genera and 4600 species being one of the most speciose groups of Diptera^1^. About 40% of the species of this family are characterized as true fruit flies since they infect a great variety of fruit producing plants and a significant number of them are considered serious agricultural pests. The *Anastrepha*, *Bactrocera*, *Ceratitis*, *Dacus* and *Rhagoletis* genera include some of the world’s most destructive insect pests, causing extensive damages in a wide variety of crops with enormous economic impact^2^.

*Ceratitis fasciventris* is an African fruit fly, which, together with *C*. *anonae*, *C*. *rosa* and the recently described *C*. *quilicii* comprise the“Ceratitis FAR complex”^3, 4^. All members of the complex are agricultural pests with *C*. *rosa* being the most aggressive one. They attack a great number of wild and cultivated plants belonging to more than 25 different families, and present greatly overlapping host range while each also has unique hosts^5, 6^. *Ceratitis fasciventris* is found mainly in eastern and western Africa sympatrically with *C*. *anonae*, while *C*. *rosa* and *C*. *quilicii* are distributed in eastern and southern Africa overlapping with each other and partially with *C*. *fasciventris* in Kenya and Tanzania^4, 6–8^. There is increasing concern about the possibility that these species could expand outside their native range similarly to the spread of *C*. *capitata* almost worldwide in the past century^2, 3, 6, 9, 10^. Populations of *C*. *rosa* and *C*. *quilicii* have been already introduced in the Indian ocean islands Mauritius and La Réunion^4, 11^ and their invasive potential is a major consideration taking into account their potential adaptation to a wider temperature range than *C*. *capitata* ^6, 12–14^ and their capability to attack fruits grown also in temperate climates (e.g. peaches and apples)^4, 15^.

Because of the high risk of their expansion, the delimitation and accurate identification of species within the FAR as well as other complexes of economic relevant tephritid pests is of crucial importance for regulatory agencies and agricultural countries. It affects the international trade and quarantine policies implemented on fruit and vegetable hosts with significant economic impact^16^. Besides, the clarification of genetic relationships among the different entities of species complexes is critical for the effective development and application of environmental friendly methods for their control, such as the Sterile Insect Technique (SIT)^17, 18^. Within the FAR complex the species boundaries are obscure and have been the object of research and discussion for some time.The members of the complex are almost identical morphologically. Their identification has been based on differences in the setal pattern of the adult male mid legs, while females are almost indistinguishable^4, 19, 20^. Due to the absence of clear diagnostic morphological features a number of molecular approaches, including mitochondrial markers and the ITS1 sequence, have been used unsuccessfully to clearly resolve the limits among the morphospecies of the complex^3, 21–23^. A more recent microsatellite analysis revealed the presence of five genotypic clusters within the complex: two (R1, R2) representing populations that were at the time considered as *C*. *rosa*, two (F1, F2) for *C*. *fasciventris* and one for *C*. *anonae* ^8^, thus complicating further the phylogenetic relationships and species discrimination in the FAR complex.

Recent studies have shown that efforts to resolve complex species status require multidisciplinary approaches^24, 25^, well-characterized material and extended sampling^26–28^. An example is the study of the members of the *Bactrocera dorsalis* complex, *B*. *dorsalis s*.*s*., *B*. *papayae*, *B*. *philippinensis*, *B*. *invadens* and *B*. *carambolae*, for which morphological/morphometric, behavioral/sexual compatibility, chemoecological, molecular/genetic and cytogenetic data lead to the synonymization of the four members, maintaining *B*. *carambolae* as a discrete entity^29^. Pluralistic approaches have been also followed for species delimitation within species complexes of other Tephritidae genera, such as the *Anastrepha fraterculus* complex^18, 30–35^. In this context, the issue of the number and the limits of species within the FAR complex has been recently addressed by an integrative approach on the basis of: (i) adult and larvae morphology^36, 37^, (ii) wing morphometrics^38^, (iii) microsatellite analysis^8^, (iv) cuticular hydrocarbons^39^, (v) pheromones^40^, (vi) developmental physiology^41^ and (vii) geographical and altitudinal distribution^42^. The above analysis supported the species identity of *C*. *anonae* ^36^. For *C*. *rosa*, it was proposed that it consists of two separate entities (R1 and R2) that should be considered as different species: *C*. *rosa* representing group R1 and the newly described species *C*. *quilicii* representing group R2^4, 36^. Although two entities (F1 and F2) were identified for *C*. *fasciventris* as well, the available data are insufficient to support their separation in two different species^36^.

The above analysis, although incorporating a volume of data from several disciplines, lacks chromosome/cytogenetic and mitochondrial evidence. Cytogenetic analyses can be proven valuable in efforts to resolve phylogenetic relationships and species boundaries among closely related species. Many years of cytogenetic studies in *Drosophila* and mosquitoes have shown that chromosomal rearrangements (CRs), especially inversions, play a causative role in speciation and they can be used as interspecific phylogenetic markers^43–48^. Modern genomic support the above observations and propose that CRs enhance speciation through the restriction of recombination within and near inverted regions resulting in restriction of gene flow^49–58^.

In Tephritidae flies, mitotic karyotypes have been used to distinguish between different members of species complexes based on differences of the sex chromosomes^33, 35, 59–64^. Polytene chromosome maps are available for 11 tephritid species belonging to five genera^65–79^. Comparative analyses among most of them revealed specific CRs that are diagnostic at the genus, subgenus and species level^70, 75–78, 80^, supporting the possible involvement of CRs in speciation.

The mitochondrial DNA (mt DNA) is considered a very useful molecular marker for phylogenetic analyses as it can be informative at a variety of taxonomic levels^81, 82^. Partial mitochondrial gene sequences have been extensively used for inferring phylogeny among species of the Tephritidae family^83–94^, and within the *Ceratitis* genus, in particular^3, 22, 23^. However, the use of complete mitochondrial genome sequences, facilitated by the continuously increasing number of them in the databanks, has become a preferable approach for phylogenetic and molecular systematic studies in several insect groups^95–103^ including Tephritidae^104–110^. When the issue is the discrimination of closely related species for quarantine and management applications the availability of complete mitogenome data is particularly valuable, as it can allow identification of the most informative diagnostic markers/sequences through intra and inter-specific comparative analysis^106^.

In the current study, we present the mitotic karyotype, a detailed salivary gland polytene chromosome analysis, as well as the complete mitochondrial genome sequence of *C*. *fasciventris*. This is the first description of the mitotic and polytene chromosomes, as well as the first complete mitogenome sequence from a member of the *Ceratitis* FAR complex and only the second available for the whole genus, after the model species, *C*. *capitata*. Both cytogenetic and mitochondrial information provided can be used as reference for comparative studies towards species delimitation and resolution of phylogenetic relationships within this species complex, but also among other species of the *Ceratitis* genus.

## Results and Discussion

### Mitotic chromosomes

**Figure 1:**
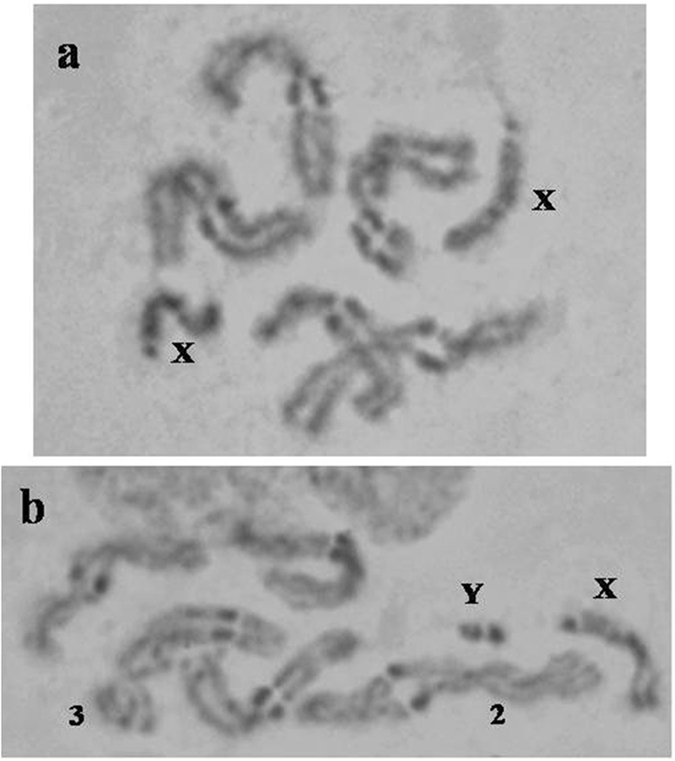
Mitotic karyotype of *Ceratitis fasciventris*. (**a**) Female karyotype. The X chromosomes are indicated. (**b**) Male karyotype. The autosomes 2 and 3 and the X and Y chromosomes are indicated. Chromosomes were stained with Giemsa.

The mitotic karyotype of *Ceratitis fasciventris* consists of six pairs of chromosomes: five pairs of meta- or sub-metacentric autosomes and one pair of sex chromosomes (Fig. 1). According to the labeling system used for the Mediterranean fruit fly (medfly), *C*. *capitata* (Wiedemann)^67^, the sex chromosomes are designated as the first pair of the mitotic karyotype, while the five autosomes are labeled from 2 to 6, in order of descending size. The first autosome pair (2) of *C*. *fasciventris *is easily distinguished due to its larger size. The remaining four autosomes are almost equal in size. One of them is clearly submetacentric and has been designated as autosome pair 3 in accordance to *C*. *capitata*, while the other three cannot be reliably distinguished (Fig. 1b). The sex chromosomes are identified as the heteromorphic pair (XY) of heavily stained chromosomes in the mitotic complement (Fig. 1b). This karyotype is consistent with the karyotype of most Tephritidae species analyzed so far^59–61, 65, 67, 68, 70, 71, 73–77, 79, 111^.

The above described mitotic karyotype of *C*. *fasciventris* is quite similar to the karyotype of *C*. *capitata* ^66, 67^. The most apparent difference between the two species is the size of the sex chromosomes. In *C*. *capitata*, the X chromosome is about the same size with the largest autosome, while the Y is the shorter chromosome of the mitotic complement. On the contrary, in *C*. *fasciventris* the X chromosome is shorter than any autosome, while the Y chromosome is significantly smaller with its length reaching about ¼ of the length of the X. Size and shape variation of the sex chromosomes is common among tephritids and, based on their highly heterochromatic nature, it could be attributed to the accumulation or loss of heterochromatin^30, 31, 33, 35, 59–61, 63–65, 67, 68, 70–79, 111–116^.

### Polytene chromosomes

**Figure 2:**
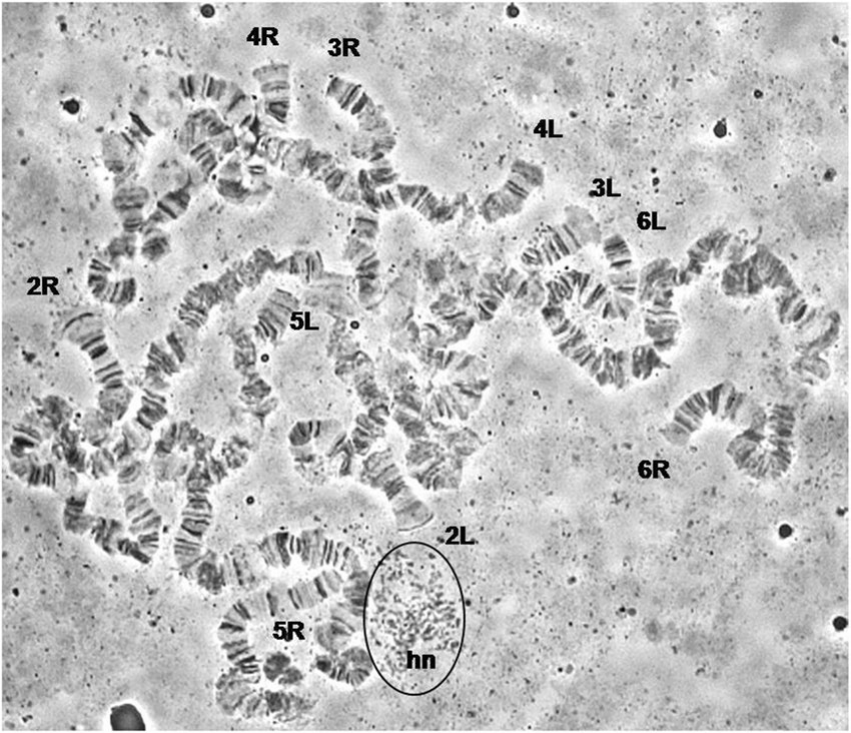
A polytene nucleus of *Ceratitis fasciventris*. The telomeres of the polytene elements are indicated. The heterochromatic network (hn) corresponding to the sex chromosomes is circled.

**Figure 3:**
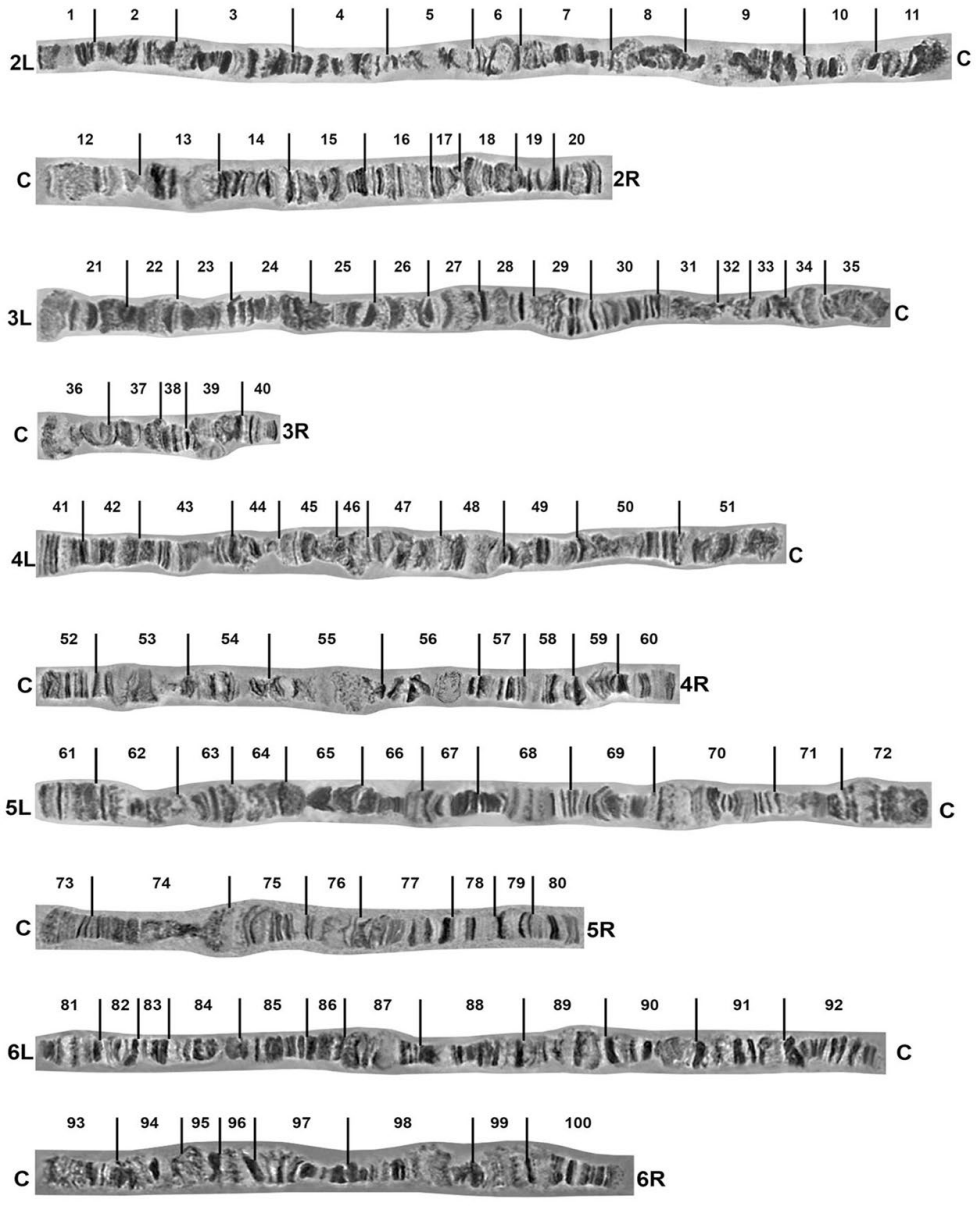
Photographic polytene chromosome map of *Ceratitis fasciventris*. C indicates the centromere.

The analysis of the salivary gland polytene chromosomes of *C*. *fasciventris* showed that the polytene complement consists of five long, well banded chromosomes (10 polytene arms) corresponding to the five autosomes. The sex chromosomes are under-replicated in polytene tissues, do not form discrete polytene elements and are apparent as a heterochromatic network (Fig. 2). No typical chromocenter exists; the two arms of each individual chromosome are loosely connected or can be found separated from each other. The polytene chromosomes of *C*. *fasciventris* were numbered from 2 to 6 and divided into sections from 1 to 100 based on their banding pattern similarities to *C*. *capitata*. For each polytene chromosome, the longer arm is designated as left (L) and the shorter one as right (R) (Fig. 3).

**Figure 4:**
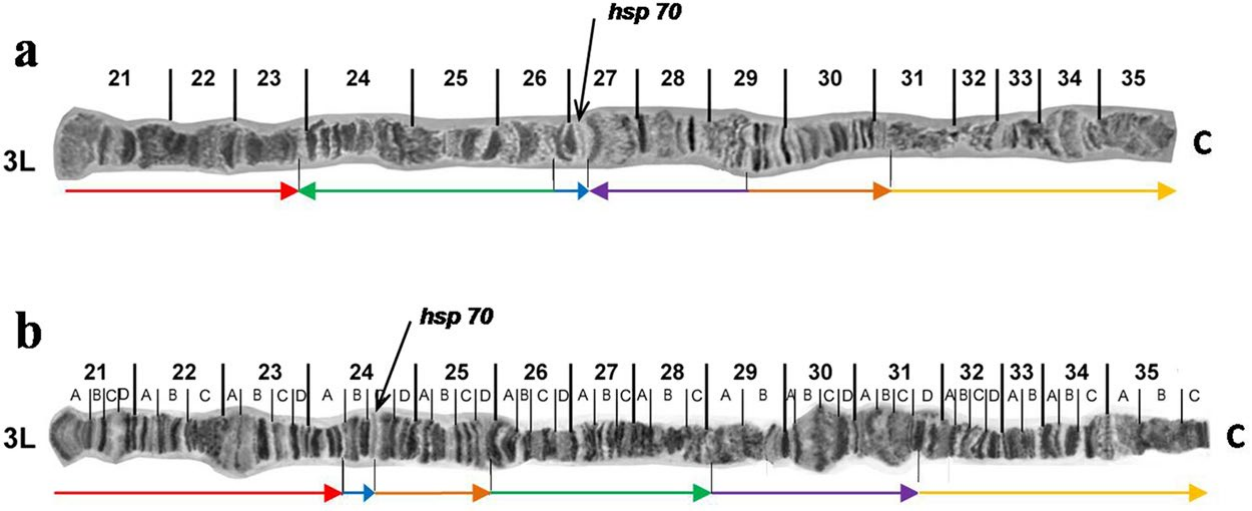
Comparison of the 3L polytene chromosome arms of *Ceratitis fashiventris* and *Ceratitis capitata*. (**a**) The 3L polytene chromosome arm of *C*. *fasciventris*; (**b**) The 3L polytene chromosome arm of *C*. *capitata*. Horizontal arrows of the same color show the position and relative orientation of the putative corresponding chromosome segments between the two species. Vertical black lines underneath the chromosomes show the proposed breakpoints. Black arrows indicate the hybridization site of the *hsp70* probe in each species. C indicates the centromere.

**Figure 5:**
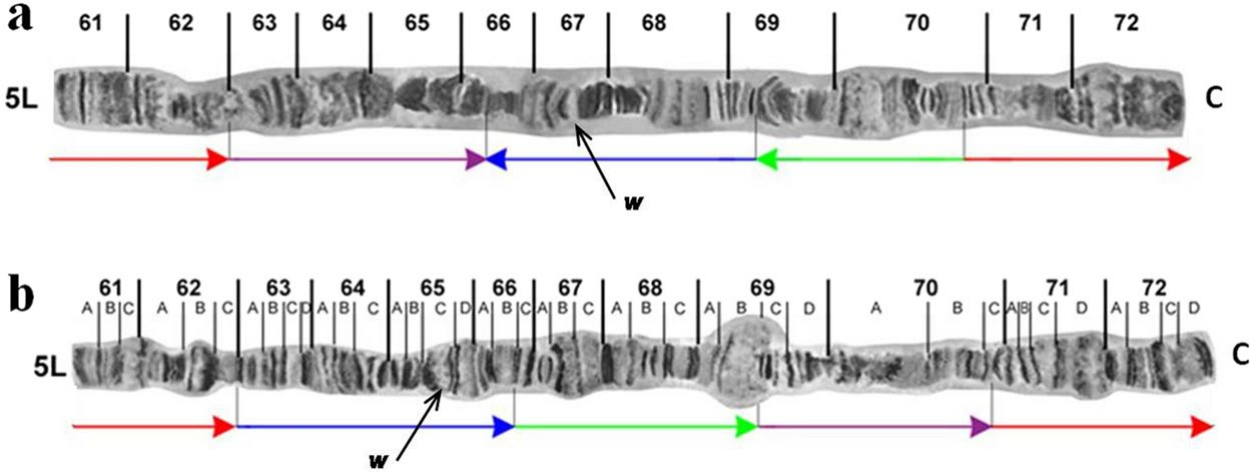
Comparison of the 5L polytene chromosome arms of *Ceratitis fashiventris* and *Ceratitis capitata*. (**a**) The 5L polytene chromosome arm of *C*. *fasciventris*; (**b**) The 5L polytene chromosome arm of *C*. *capitata*. Horizontal arrows of the same color show the position and relative orientation of the putative corresponding chromosome segments between the two species. Vertical black lines underneath the chromosomes show the proposed breakpoints. Black arrows indicate the hybridization site of the *w* probe in each species. C indicates the centromere.

Detailed comparison of the polytene chromosomes of *C*. *fasciventris* to the reference map of *C*. *capitata* ^67, 117^ revealed significant similarities. Characteristic structures such as the distal ends (telomeres) and the centromeric region of each chromosome seem identical between the two species. Furthermore, extensive similarity in the banding pattern of eight out of the ten polytene arms, namely 2L, 2R, 3R, 4L, 4R, 5R, 6L and 6R, can be identified (Supplementary Figs S1–S8). However, some differences in the puffing pattern were observed, which could reflect species-specific differences, different developmental stages^118^ and/or differences at rearing conditions. Regarding chromosome arms 3L and 5L, despite the banding pattern similarity in both ends of each arm, differences can be observed in the inner parts. After thorough analysis of the banding pattern, specific chromosome segments that seem to be rearranged in comparison to *C*. *capitata* have been identified (Figs 4 and 5). These changes could have been derived from consecutive overlapping inversions resulting in both inverted and transposed chromosome fragments^119^. Comparative analyses of polytene chromosomes among several tephritids showed that CRs are restricted to specific chromosome arms, mainly to the 3L and 5L arms^70, 76–78, 80^.

### Chromosome localization of molecular markers

**Figure 6:**
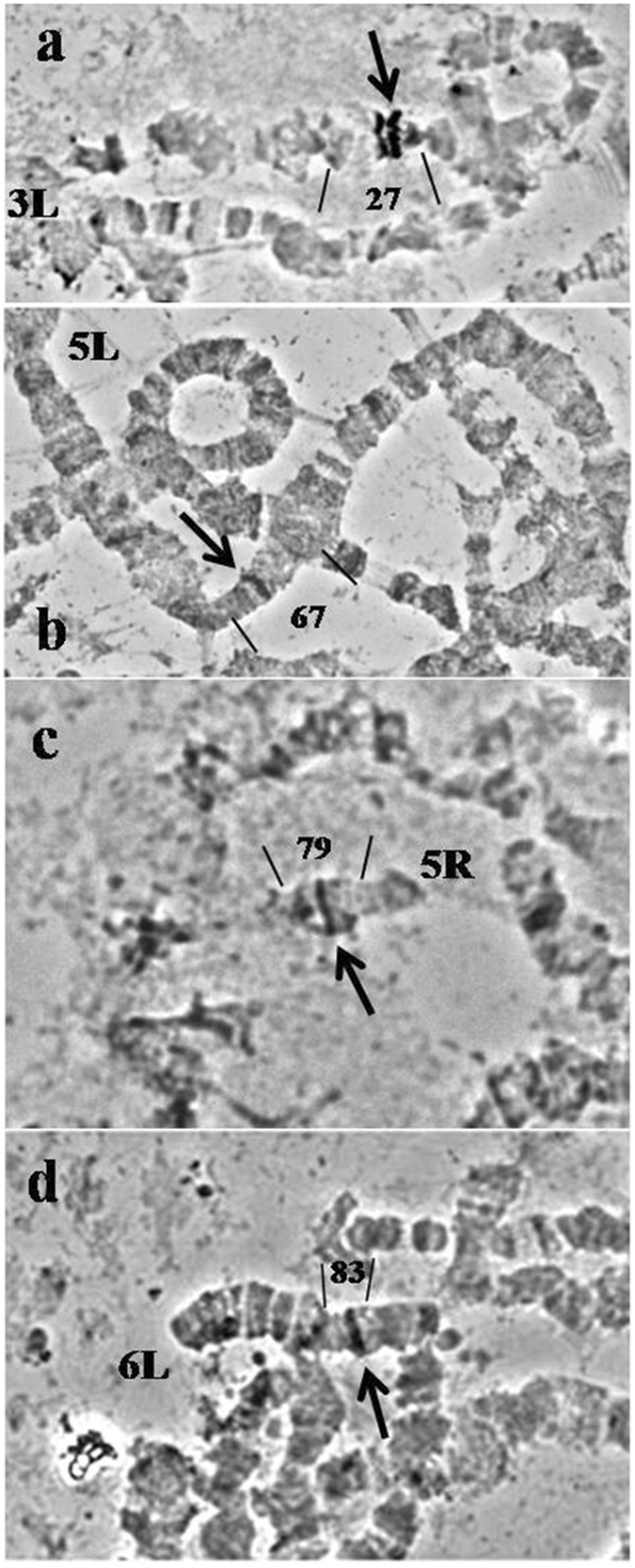
*In situ* hybridization on the salivary gland polytene chromosomes of *Ceratitis fasciventris*. (**a**) hybridization site of the *hsp70* probe; (**b**) hybridization site of the *w* probe; (**c**) hybridization site of the *sxl* probe and (**d**) hybridization site of the *st* probe. Arrows indicate the hybridization signals.

Four gene markers, namely *hsp70*, *w*, *sxl* and *st* (Table 1), were localized on the polytene chromosomes of *C*. *fasciventris* by *in situ* hybridization. Each heterologous probe gave a unique hybridization signal (Table 1 and Fig. 6): (i) the *hsp 70* specific probe on region 27 of the 3L arm (Fig. 6a), (ii) the *w* probe on region 67 of the 5L polytene arm (Fig. 6b), (iii) the *sxl* probe on region 79 of the 5R arm (Fig. 6c) and (iv) the *st* probe on region 83 of the 6L polytene arm (Fig. 6d).

**Table 1:** The hybridization probes and their localization sites on the polytene chromosomes of *Ceratitis fasciventris* and *Ceratitis capitata*.

Gene symbol	Description	Species of origin	DNA type	Localization site *C*. *fasciventris/C*. *capitata*		References
*hsp70*	The gene for the heat-shock 70 protein	*Ceratitis capitata*	genomic	**27-3L**	**24-3L **	121
*w*	The orthologue of the *D*. *melanogaster white* gene	*Bactrocera tryoni*	genomic	**67-5L**	**65-5L**	120, 147
*sxl*	The orthologue of the *D*. *melanogaster sex lethal* gene	*Bactrocera oleae*	cDNA	**79-5R**	**79-5R**	117, 148
*st*	The orthologue of the *D*. *melanogaster scarlet* gene	*Bactrocera tryoni*	genomic	**83-6L**	**83-6L**	117, 149

The four gene markers studied here have been previously mapped in *C*. *capitata* at putative corresponding polytene chromosome regions^70, 117, 120, 121^. The localization of the *sxl* and s*t* genes supports the conservation of the 5R and 6L polytene chromosome arms between the two species, as it was deduced by banding pattern similarities (Supplementary Figs S6 and S7). On the other hand, the hybridization of the *hsp70* gene at region 27 of *C*. *fasciventris*is is in discordance with the localization of the above gene at region 24 of *C*. *capitata* (Table 1 and Fig. 4), supporting the proposed rearrangements of the 3L polytene arm between the two species (Fig. 4). Similarly, the different chromosome region of the *w* locus (67 on 5L for *C*. *fasciventris*, 65 on 5L for *C*. *capitata*; Table 1 and Fig. 5) is further support for the rearrangements of the 5L chromosome arm between the two species suggested by banding pattern analysis (Fig. 5).

The banding pattern comparison and gene localization data provide evidence for the conservation of chromosome synteny between the closely related species *C*. *capitata* and *C*. *fasciventris*. However, a more thorough chromosome mapping analysis would be needed in order to confirm the above observation. The idea that the chromosome elements have maintained their basic content and identity was initially proposed for *Drosophila* species^122, 123^. Later comparative studies using biochemical and morphological markers showed a remarkable conservation of linked gene clusters among a wider phylogenetic range of higher Diptera^124–126^. *In situ* hybridization of well characterized genes on polytene chromosomes further supported the extensive conservation of linkage groups in Tephitidae^70, 117, 127–131^. On the other hand, the differences identified in the structure of the 3L and 5L polytene arms of *C*. *fasciventris* and *C*. *capitata*, are congruent with the concept that chromosome rearrangements, mainly paracentric inversions, are implicated in species differentiation in Diptera which is substantiated by comparative cytogenetic and genomic analyses in *Drosophila* and mosquitoes^46–54, 56–58^. In accordance with the above notion, comparisons among tephritids, reveal that inversions and/or transpositions on specific chromosomes, including chromosomes 3 and 5, differentiate species and support the potential value of chromosome rearrangements as phylogenetic and diagnostic markers among closely related species^70, 75–78, 80, 111^.

### Mitochondrial genome

The mitochondrial DNA of *C*. *fasciventris* was amplified and sequenced in 27 overlapping fragments. The above analysis resulted in the nucleotide sequence of the complete *C*. *fasciventris* mitogenome consisting of 16,017 bp with very high A + T (77.17%) and very low G + C (22.83%) contents. The organization of the *C*. *fasciventris* mt genome is typical to that of other tephritids studied^104, 106–110, 132–136^. It comprises 13 protein-coding, two *rRNA* (12S and 16S *rRNA*) and 22 *tRNA* genes, and one major non-coding sequence, the control region (Table 2), all of which are similar in size to their counterparts in other insects.

**Table 2:** Organization of the *Ceratitis fasciventris* mitochondrial genome.

Gene/Element	Abbreviation	Strand	Position	Size (bp)	Inter-genic spacer	Start codon	Stop codon	Codon recognized	Amino acids
*tRNA* ^*Ile*^	*I*	H	1–67	67	62			ATC	
*tRNA* ^*Gln*^	*Q*	L	130–198	69	16			CAA	
*tRNA* ^*Met*^	*M*	H	215–283	69	1			ATG	
*NADH dehydrogenase subunit 2*	*ND2*	H	285–1307	1023	4	ATT	TAA		340
*tRNA* ^*Trp*^	*W*	H	1312–1379	68	−8			TGA	
*tRNA* ^*Cys*^	*C*	L	1372–1436	65	20			TGC	
*tRNA* ^*Tyr*^	*Y*	L	1457–1523	67	−2			TAC	
*Cytochrome c oxidase subunit 1*	*COI*	H	1522–3057	1536	6	TCG	TAA		511
*tRNA* ^*Leu*^ (*UUR*)	*L*	H	3064–3129	66	5			TTA	
*Cytochrome c oxidase subunit 2*	*COII*	H	3135–3821	687	8	ATG	TAA		228
*tRNA* ^*Lys*^	*K*	H	3830–3900	71	4			AAG	
*tRNA* ^*Asp*^	*D*	H	3905–3971	67	0			GAC	
*ATP synthase F0 subunit 8*	*ATP8*	H	3972–4133	162	−7	ATT	TAA		53
*ATP synthase F0 subunit 6*	*ATP6*	H	4127–4804	678	−1	ATG	TAA		225
*Cytochrome c oxidase subunit 3*	*COIII*	H	4804–5592	789	6	ATG	TAA		262
*tRNA* ^*Gly*^	*G*	H	5599–5663	65	0			GGA	
*NADH dehydrogenase subunit 3*	*ND3*	H	5664–6017	354	2	ATA	TAA		117
*tRNA* ^*Ala*^	*A*	H	6020–6083	64	19			GCA	
*tRNA* ^*Arg*^	*R*	H	6103–6166	64	3			CGA	
*tRNA* ^*Asn*^	*N*	H	6170–6234	65	0			AAC	
*tRNA* ^*Ser*^(*AGY*)	*S*	H	6235–6302	68	0			AGC	
*tRNA* ^*Glu*^	*E*	H	6303–6370	68	18			GAA	
*tRNA* ^*Phe*^	*F*	L	6389–6455	67	0			TTC	
*NADH dehydrogenase subunit 5*	*ND5*	L	6456–8175	1720	18	ATT	T*		573
*tRNA* ^*His*^	*H*	L	8194–8258	65	12			CAC	
*NADH dehydrogenase subunit 4*	*ND4*	L	8271–9611	1341	−1	ATG	TAA		446
*NADH dehydrogenase subunit 4L*	*ND4L*	L	9611–9901	291	2	ATG	TAA		96
*tRNA* ^*Thr*^	*T*	H	9904–9968	65	0			ACA	
*tRNA* ^*Pro*^	*P*	L	9969–10034	66	2			CCA	
*NADH dehydrogenase subunit 6*	*ND6*	H	10037–10561	525	−1	ATT	TAA		174
*Cytochrome b*	*CYTB*	H	10561–11697	1137	−2	ATG	TAG		378
*tRNA* ^*Ser*^(*UCN*)	*S*	H	11696–11762	67	15			TCA	
*NADH dehydrogenase subunit 1*	*ND1*	L	11778–12717	940	9	ATT	T*		313
*tRNA* ^*Leu*^(*CUA*)	*L*	L	12727–12792	66	0			CTA	
*16S rRNA*	*16S*	L	12793–14133	1341	0				
*tRNA* ^*Val*^	*V*	L	14134–14205	72	0			GTA	
*12S rRNA*	*12S*	L	14206–14993	788	0				
Control region	CR		14994–16017	1024	0				

*TAA stop codon is completed by the addition of 3′ A residues to mRNA.

### Protein coding genes

The location of the 13 protein coding genes (PCGs) was determined by the identification of initiation and termination signals as well as by sequence comparisons to the respective genes of *C*. *capitata*. Nine of them are encoded by the H strand, while *ND1*, *ND4*, *ND4L* and *ND5* are encoded by the L strand (Table 2). Only six protein coding genes (*COII*, *ATP6*, *COIII*, *ND4*, *ND4L*, *CYTB*) begin with the canonical initiation codon ATG, five of them (*ND2*, *ATP8*, *ND5*, *ND6* and *ND1*) initiate with ATT, while for *ND3* the first codon is ATA and for *COI* it is TCG (Table 2), similarly to other Tephritidae species^104, 106–108, 110, 132, 133^. In *C*. *fasciventris*, two of the protein coding genes (*ND5* and *ND1*) possess an incomplete termination codon (T) (Table 2). This is common in animal mitochondrial DNA; the stop codon is likely completed by post-transcriptional polyadenylation^137^.

The overlapping of seven nucleotides between *ATP8* and *ATP6* genes is the longest observed between protein coding genes of *C*. *fasciventris* and is also common (presenting variable size) among tephritids^104, 106, 132, 133^. Overlapping restricted to one or two nucleotides can also be observed between *ATP6* and *COIII*, *ND4* and *ND4L*, *ND6* and *CYTB* and *CYTB* and *tRNA* ^*Ser*^ (Table 2). However, in these cases one could assume an incomplete stop codon for the preceding gene, which could mean lack of overlap with the following one.

### RNA genes

The *C*. *fasciventris* 16S *rRNA* and 12S *rRNA* genes consist of 1,341 (positions: 12,793–14,133) and 788 (positions: 14,206–14,993) nucleotides, respectively (Table 2). As in other insects, these genes are located in the L strand at the end of the mtDNA molecule between the gene for *tRNA* ^*Leu*^ (CUA) and the control region, and are separated by the *tRNA* ^*Val*^ gene. Dispersed among the protein-coding and the *rRNA* genes there are 22 *tRNA* genes; 14 on the H and 8 on the L strand. Their sizes range from 64–72 nucleotides (Table 2) and are predicted to fold into the expected cloverleaf secondary structures.

### Non-coding regions

Following the rule for animal mitochondrial DNA, the mt genome of *C*. *fasciventris* contains only one long non-coding region with very high A + T content (90.24%) that regulates replication and transcription, the control region. It is located between the 12S*rRNA* and the *tRNA* ^*Ile*^ genes and its length is 1024 bp (positions 14,994–16,017; Table 2), within the range of the respective region in other Tephritidae species. At the 5′ end of the control region (positions 15,947–15,973) a poly(T) followed by a [TA(A)n]-like stretch can be observed. This feature is conserved among insect species and has been proposed to play a role in the control of transcription and/or replication^107, 110, 133, 138^. Two motifs, TTAAATTAATAATTAT and TATTTTTATTTTTAAATT, were found to be tandemly repeated three (positions 15,263–15,315) and two times (positions 15,871–15,909), respectively. Tandem repeats have been identified in the control regions of other Tephritidae species, as well^107, 133^.

The *C*. *fasciventris* mitogenome contains 20 intergenic spacers (IGS) of 232 bp total length (Table 2). The longest one (62 bp) is located between *tRNA* ^*Ile*^ and *tRNA* ^*Gln*^, as is the case in *B*. *zonata* ^107^. In *C*. *capitata*, an IGS is present at the respective position but it is not the longer one. In the latter species, the longer IGS (46 bp) is found between *tRNA* ^*Gln*^ and *tRNA* ^*Met*^ ^132^ similarly to several *Bactrocera* species^106, 108, 109^. Quite long IGS in species of the Tephritidae family are also present between *tRNA* ^*Cys*^ and *tRNA* ^*Tyr*^, which is able to form a secondary structure and between *tRNA* ^*Arg*^ and *tRNA* ^*Asn*^ ^104, 106, 108–110, 132–134^. The second longest IGS in *C*. *fasciventris* lies between *tRNA* ^*Cys*^ and *tRNA* ^*Tyr*^; however it is only 20 bp long and cannot form a stem loop. On the other hand, IGS separating *tRNA* ^*Arg*^ and *tRNA* ^*Asn*^ is only 3 bp long. It has been observed thatspecific IGS (the ones between *tRNA* ^*Glu*^ and *tRNA* ^*Phe*^, *ND5* and *tRNA* ^*His*^, *tRNA* ^*Ser*^ and *ND1* and *ND1* and *tRNA* ^*Leu*^) are conserved in size and sequence among several tephritids and have their counterparts in the control region^107, 133^. This is also valid for *C*. *fasciventris*.

### Sequence comparisons with C. capitata and other Tephritidae species

The *C*. *fasciventris* mitogenome is the second complete mt DNA sequence of the *Ceratitis* genus analyzed, with the first one being that of *C*. *capitata* ^132^. The mitochondrial genomes of these two species are highly similar both in terms of organization and structure as well as of sequence similarity. The overall sequence identity is 92.11%, while identity of the PCGs is 92.25% (Table 3). In comparison to other tephritid mitogenomes, the *C*. *fasciventris* mtDNA presents the lowest sequence identity with *B*. *minax* (79.18% for complete mitogenome; 79.31% for PCGs). The latter species presents the lowest identity percentages in comparison with any other tephritid, even the ones of the same genus, both in the complete mitosequence, as well as in the PCGs sequence (Table 3).

**Table 3:** Identity percentage matrix among 19 Tephritidae mitochondrial genome sequences.

	fas	cap	are	cor	car	dor	try	zon	mel	lat	umb	ole	min	cau	cuc	dia	scu	tau	lon
**fas**	—	92.11	84.05	84.40	84.92	84.94	84.21	84.57	84.83	82.91	82.53	84.19	79.18	84.87	84.30	85.53	85.04	85.63	82.69
**cap**	92.25	—	84.03	84.73	84.89	84.89	84.27	84.67	85.04	83.07	82.54	84.36	78.90	84.59	84.13	85.59	84.67	85.56	82.97
**are**	83.77	83.68	—	90.12	91.26	91.15	90.76	90.86	90.52	88.08	87.92	86.47	81.29	85.55	84.57	86.08	86.09	85.96	83.47
**cor**	84.65	84.97	90.09	—	91.62	91.64	90.53	94.09	90.58	87.53	87.24	86.65	81.06	85.75	84.98	86.37	85.81	86.07	83.57
**car**	84.70	84.64	90.13	91.59	—	98.83	92.22	92.30	91.61	88.34	88.41	87.01	81.93	85.97	85.67	87.11	86.70	86.77	84.04
**dor**	84.72	84.62	89.98	91.57	98.60	—	92.12	92.29	91.68	88.35	88.33	87.05	82.06	85.99	85.70	87.14	86.59	86.81	84.00
**try**	83.87	83.88	89.73	90.38	91.12	90.99	—	91.32	90.98	88.21	88.03	86.50	81.18	85.60	85.10	86.36	86.17	86.20	83.74
**zon**	84.62	84.68	90.14	93.71	91.46	91.42	90.52	—	91.34	87.98	87.69	86.88	81.10	85.65	85.16	86.71	86.10	86.19	83.70
**mel**	84.57	84.77	89.42	90.50	90.76	90.86	89.84	90.69	—	87.80	87.71	86.64	81.14	85.79	84.99	86.74	86.22	86.14	83.63
**lat**	82.19	82.36	86.77	87.26	86.93	86.97	87.04	87.06	86.66	—	86.07	85.38	80.40	84.46	83.60	85.03	84.71	84.86	82.68
**umb**	81.91	81.81	86.59	86.73	87.15	87.08	86.75	86.65	86.36	84.58	—	84.75	80.35	83.48	82.89	83.88	83.86	83.75	82.24
**ole**	83.89	83.97	85.63	86.31	85.90	85.95	85.39	86.26	85.54	84.33	83.41	—	81.19	85.93	84.74	86.38	86.12	86.08	83.49
**min**	79.31	79.04	80.97	81.27	81.84	81.90	80.82	81.13	80.72	79.86	79.86	80.70	—	81.17	80.69	81.26	81.29	81.24	80.03
**cau**	84.57	84.15	84.66	85.42	84.91	84.91	84.59	85.06	84.99	83.50	82.25	84.97	81.05	—	87.60	92.52	92.52	89.23	85.07
**cuc**	84.28	83.89	83.79	84.72	84.89	84.91	84.30	84.77	84.16	82.75	81.87	83.96	81.86	86.87	—	88.63	88.43	94.58	84.06
**dia**	85.28	85.34	85.21	86.16	86.10	86.15	85.44	86.32	85.92	84.05	82.49	85.43	81.12	91.48	87.77	—	94.40	90.66	85.51
**scu**	84.74	84.17	85.06	85.44	85.60	85.41	85.13	85.48	85.12	83.64	82.44	85.17	81.32	91.35	87.49	93.36	—	90.07	85.25
**tau**	85.13	84.84	84.92	85.52	85.67	85.67	85.06	85.50	85.02	83.67	82.34	84.88	81.72	88.01	94.32	89.37	88.54	—	85.38
**lon**	81.95	82.14	82.14	82.86	82.66	82.63	82.44	82.81	82.39	81.32	80.78	82.23	79.65	83.63	83.14	83.98	83.71	83.97	—

In the upper part the identities of complete mitochondrial DNA sequences are given. In the lower part the identities of the concatenated protein coding gene sequences are given. Species abbreviations and GenBank accession numbers of the mtDNA sequences used are given in Supplementary Τable S1.

**Figure 7:**
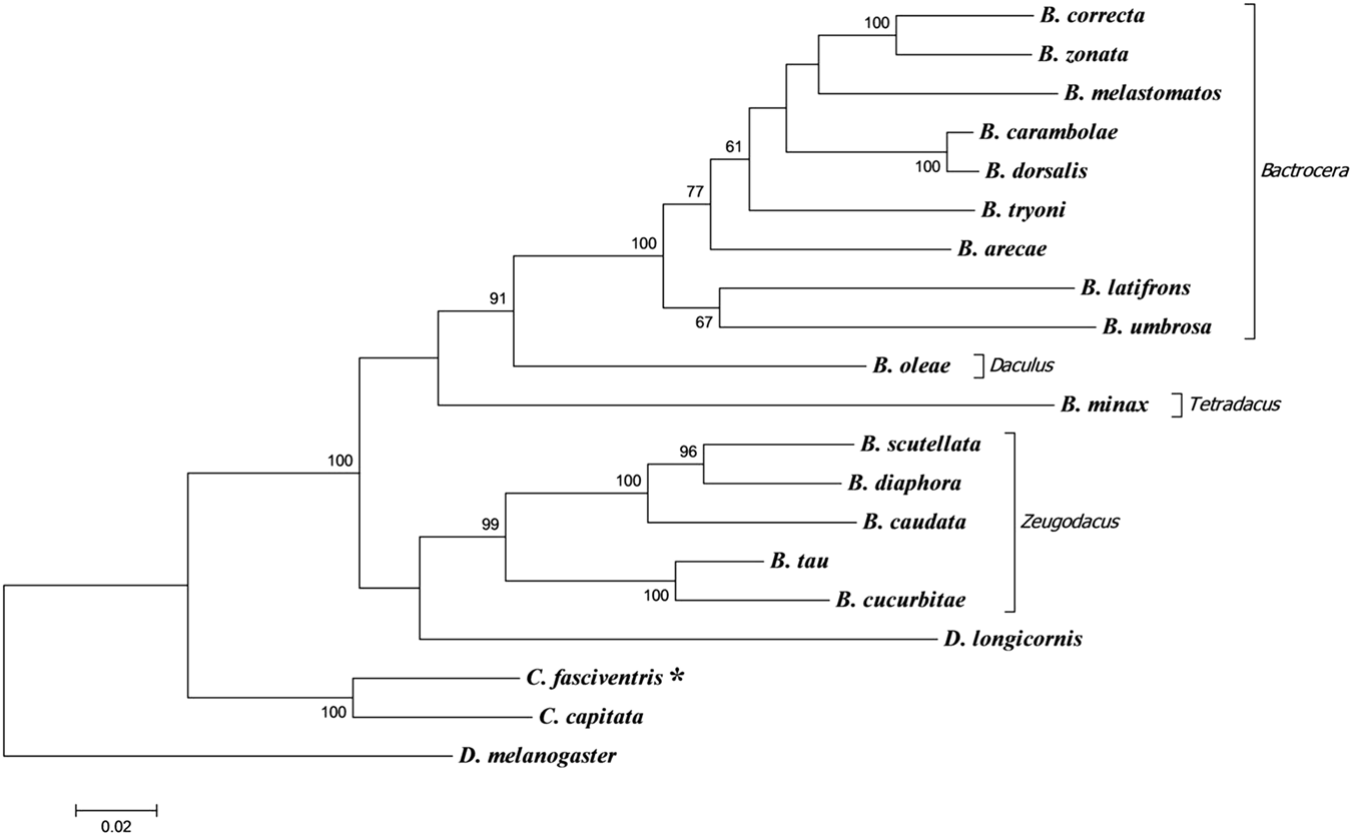
Molecular phylogenetic analysis by Maximum Likelihood method. Tree based on 13 protein coding genes from complete mitochondrial genome sequences of 19 Tephritidae species. The tree is drawn to scale, with branch lengths measured in the number of substitutions per site. Asterisk indicates the *Ceratitis fasciventris* sequence analyzed in the present study. Clustering based upon subgenera for the *Bactrocera* genus is indicated by bars along the right hand side of the phylogenetic tree. *Drosophila melanogaster* was used as out group to root the tree. Numbers at nodes are for bootstrap percentages from 1000 replicates; only the ones higher than 50 are presented. The GenBank accession numbers of the mtDNA sequences used are given in Supplementary Table S1.

The phylogenetic analysis based on the concatenated sequences of the 13 PCGs of 19 Tephritidae species places the currently analyzed *C*. *fasciventris* sequence in the same clade with the sequence of *C*. *capitata* (Fig. 7) confirming the closer relationship of the two species^22, 139^. Furthermore, species of the *Zeugodacus* subgenus are grouped separately from all other *Bactrocera* subgenera in a sister clade to *Dacus* (Fig. 7), supporting the recent suggestion of raising *Zeugodacus* to the genus level, as well its closer phylogenetic relationship to *Dacus* in comparison to B*actrocera* ^88, 92, 94, 110, 140^.

## Conclusions

In the crucial issue of species boundaries within complexes of destructive insect pests, a multidisciplinary approach is considered the best way for reaching solid results and reliable species discrimination^24, 25, 29^. To this direction, cytogenetic and complete mitogenome analysis can provide important information and potentially reveal species diagnostic characters. The mitotic karyotype and polytene chromosome maps of *C*. *fasciventris* are the first presented for the FAR complex and the second for the genus *Ceratitis*. The comparison and the differences identified with the mitotic and polytene chromosomes of *C*. *capitata* proves the cytogenetic information on *C*. *fasciventris* is valuable for comparative analysis possibly providing important insight in the genetic relationships of the species of the FAR complex, as well as among other species of the *Ceratitis* genus. Likewise, the first complete mitochondrial sequence of a member of the FAR complex can be used as reference for future sequence comparisons among FAR species, aiming to reveal polymorphic mitochondrial regions suitable for the development of molecular diagnostic markers. The fact that the insects used originate from a well-characterized established colony eliminates possible problems resulting from sampling and species misidentification^80^ and supports the use of both cytogenetic and mitochondrial present results as reference material.

## Methods

### Specimens and rearing conditions

Insects used in the present study came from a *C*. *fasciventris* colony maintained at the FAO-IAEA Insect Pest Control Laboratory (IPCL), Seibersdorf Austria. The colony was established in the summer of 2013 from pupae found in field infested *Citrus* sp. fruits from Kenya. Male adults were identified as belonging to the F2 group of *C*. *fasciventris*, based on the coloration and setal ornamentation of the mid tibia^36^. Identified adult voucher specimens of the colony are preserved at the entomology collections of the Royal Museum for Central Africa (Tervuren, Belgium).

Rearing was accomplished by keeping adults in groups of 300–500 individuals, in 2-side fine-mesh covered, rectangular cages (50 × 30 × 20 cm). Adults had ad libitum access to water and adult diet consisting of yeast hydrolysate and sugar at 1:3 ratio. For oviposition, females were provided with bananas, which were pin punctured with 50–70 holes to serve egg deposit. The oviposited bananas were placed over a thin sawdust layer, in plastic trays. The fully developed 3^rd^ instar larvae that exited the bananas and pupated in the sawdust were collected by sieving. The colony was reared under controlled temperature, humidity and light conditions (22 °C, 65 ± 2% RH, 14 h L: 10 h D).

### Mitotic chromosome preparations

Spread chromosome preparations were made from nerve ganglia of third instar larvae^67, 111^. Brain tissue was dissected in 0.7% NaCl solution. The material was transferred to 1% sodium citrate on a well slide for at least 15 min and in fresh fixation solution (methanol/acetic acid 3:1) for 3 min. Samples were transferred to a small drop of 60% acetic acid and dispersed using a micropipette. The cell suspension was dried by laying it on a clean slide placed on a hotplate (40–45 °C). Chromosomes were stained with Giemsa solution (5% Giemsa in 10 mM phosphate buffer, pH 6.8). Chromosome slides were analyzed at 100× magnification, using a phase contrast microscope (Leica DMR), and photographs were taken using a CD camera (ProgResCFcool; Jenoptik Jena Optical Systems, Jena, Germany). More than 20 chromosome preparations representing 20 individual larvae and at least 10 well spread nuclei per preparation were analyzed.

### Polytene chromosome preparations

Polytene chromosome preparations were made from well fed third-instar larvae or 1–2 days old pupae. Larvae were dissected in 45% acetic acid and salivary glands were carefully transferred to 3 N HCL on a depression slide for 1 min. Glands were fixed in glacial acetic acid:water:lactic acid (3:2:1) for about 5 min before stained in lacto-acetic-orcein for 5–7 min^67, 111^. Early pupae were dissected in Ringer’s solution and the salivary glands were transferred to 45% acetic acid for 2–3 min and post-fixed in 1 N HCL for 2 min. The material was passed through lacto- acetic acid (80% lactic acid:60% acetic acid, 1:1) and stained in lacto-acetic-orcein for 10–20 min. Excess stain was removed by washing the glands in lacto-acetic acid^69, 111^. For best chromosome preparations, each salivary gland was cut in two pieces, and each piece was squashed for one preparation. More than 200 chromosome slides (representing at least 150 single larvae or pupae) were prepared and observed at 63× and 100× objectives in a phase contrast microscope (Leica DMR). At least 200 well spread nuclei and/or isolated chromosomes were selected for analysis and photographed using a digital camera (see above). Selected photographs for each chromosome arm showing the best morphology were used for the construction of the *C*. *fasciventris* polytene chromosome maps, as well as for the comparison with the available maps of *C*. *capitata* ^117^.

### *In situ* hybridization

Polytene chromosome preparations for *in situ* hybridization were made from salivary glands of late third instar larvae or young pupae (1–2 day old)^128^. Four heterologous gene sequences, namely *hsp70*, *w*, *sxl* and *st* were used as probes (Table 1). Labeling was performed using the DIG-High Prime and detection using the Anti-Digoxigenin-AP, Fab fragments and the NBT/BCIP Stock Solution, all purchased by ROCHE, Mannheim, Germany. Hybridization was performed at 60 °C for the *hsp70*, *sxl* and *st* probes and at 52 °C for the *w* probe. Hybridization and detection procedures were performed as previously described^111, 128^. Four to five preparations were hybridized with each probe, and at least ten well spread nuclei per preparation were observed at 63x or 100x magnification with a Nikon Eclipse 80i or a Leica DMR phase contrast microscope, respectively. Photographs were captured using a Nikon DS-5 M-U1 (63x) or a Jenoptik ProgRes (100x) CCD camera.

### DNA isolation and mitochondrial genome amplification and sequencing

Total genomic DNA was extracted from single flies, using the CTAB protocol^141^.

The complete mtDNA sequence was obtained from a single specimen by standard PCR amplifications using: (i) heterologous primers that were designed based on the mitochondrial sequences of *Ceratitis capitata* and *Bactrocera dorsalis* (Supplementary Table S1) and (ii) homologous primers that were designed based on the sequences determined by the analysis in this study. In total 27 pairs of primers amplifying overlapping fragments were designed by the Oligoexplorer and Oligoanalyzer programs (Supplementary Table S2). The reaction mixture (25 μl) contained 1X PCR buffer, 1.5 mM MgCl_2_, 0.2 mM of each dNTP, 0.5 μM of the appropriate primers and 1 U *Taq* polymerase (BIOTAQ, BIOLINE). Approximately 30 ng of template DNA was used in each reaction. PCR reactions comprised an initial denaturation at 94 °C for 3 min, followed by 40 cycles of 45 min denaturation at 94 °C, 30 sec primer annealing at 49–56 °C and 1 min DNA chain extension at 72 °C, for standard PCR. This was followed by a final extension at 72 °C for 7 min. The resulting PCR products were analyzed in a 1.0% agarose gel stained with ethidium bromide. PCR products were purified by the Nucleospin Gel and PCR Clean up kit (Macherey Nagel, Germany).

Sequencing reactions were performed by Macrogen Europe (Amsterdam, The Nederlands). The full-length mtDNA sequence was assembled using EMBOSS Merger^142^.

### Sequence analysis

Nucleotide sequence analysis was performed using the programs BLASTn (www.ncbi.nlm.nih.gov) and ClustalOmega (www.ebi.ac.uk). The regions of the protein-coding, *rRNA*, and *tRNA* genes were initially identified by comparison with the corresponding known sequences of *C*. *capitatα* (Supplementary Table S1). Furthermore, the 13 mitochondrial protein-coding genes were defined by the presence of initiation and stop codons whereas the 22 *tRNA*s were checked for their capability to fold into cloverleaf secondary structures and the presence of specific anticodons by tRNAscan-SE (http://lowelab.ucsc.edu/tRNAscan-SE/)^143^ and MITOS (http://mitos.bioinf.uni-leipzig.de/index.py)^144^. Repeats in the control region were found by the “Tandem Repeat Finder” program (http://tandem.bu.edu/trf/trf.html)^145^.

Phylogenetic analysis based on the concatenated protein coding gene sequences from available Tephritidae mitogenomes (one for each species) (Supplementary Table S1) was performed using MEGA 7.0^146^. Multiple sequence alignment (11,242 positions total length) was constructed by ClustalW using default parameters. Phylogenetic trees were inferred by the Maximum Likelihood (ML) method based on the General Time Reversible (GTR) model with 1000 bootstrap replicates.

### Data Availability

All data generated or analysed during this study are included in this published article (and its Supplementary Information files).

## Electronic supplementary material

Supplementary Information
