# Supplementary material for: The chromosomes and the mitogenome of Ceratitis fasciventris (Diptera: Tephritidae): two genetic approaches towards the Ceratitis FAR species complex resolution
Source: Sci Rep. 2017 Jul 7;7:4877. doi: 10.1038/s41598-017-05132-3 (PMC5501848; doi:10.1038/s41598-017-05132-3)

Supplementary Information

The chromosomes and the mitogenome of *Ceratitis fasciventris* (Diptera: Tephritidae): two genetic approaches towards the *Ceratitis* FAR species complex resolution

Elena Drosopoulou[1*], Christina Pantelidou[1], Angeliki Gariou-Papalexiou[2], Antonios A. Augustinos[2,3], Tatiana Chartomatsidou[1], Georgios A. Kyritsis[3], Kostas Bourtzis[3], Penelope Mavragani-Tsipidou[1], Antigone Zacharopoulou[2]

[1]Department of Genetics, Development and Molecular Biology, School of Biology, Faculty of Sciences, Aristotle University of Thessaloniki, Thessaloniki, Greece

[2]Department of Biology, University of Patras, Patras, Greece

[3]Insect Pest Control Laboratory, Joint FAO/IAEA Divission of Nuclear Techniques in Food and Agriculture, Seibersdorf, Vienna, Austria

[*]Corresponding author
Elena Drosopoulou
Department of Genetics, Development and Molecular Biology,
School of Biology, Faculty of Sciences, Aristotle University of Thessaloniki (AUTH),
GR-54124, Thessaloniki, Greece.
Tel: + 30 2310 998291, Fax: + 30 2310 998333
e-mail: edrosopo@bio.auth.gr

Supplementary Table S1. List of the complete mtDNA sequences used in the present study. Species names, abbreviation used, GenBank accession numbers and references are given.

Species	Abbreviation	GenBank Accession number	Reference
Ceratitis fasciventris	fas	KY436396	*Present study*
Ceratitis capitata	cap	AJ242872	*Spanos et al. 2000*
Bactrocera arecae	are	NC_028327	*Yong et al. 2015*
Bactrocera correcta	cor	JX 456552	*Liu et al. 2015*
Bactrocera carambolae	car	NC_009772.1	-
Bactrocera dorsalis	dor	NC_008748	*Yu et al. 2007*
Bactrocera tryoni	try	HQ130030	*Nardi et al. 2010*
Bactrocera zonata	zon	NC027725	*Choudharry et al. 2015*
Bactrocera melastomatos	mel	NC_029467	*Yong et al. 2016*
Bactrocera latifrons	lat	NC_029466	*Yong et al. 2016*
Bactrocera umbrosa	umb	NC_029468	*Yong et al. 2016*
Bactrocera oleae	ole	AY210702	*Nardi et al. 2003*
Bactrocera minax	min	HM776033	*Zhang et al. 2014*
Bactrocera caudata	cau	KT 625492	-
Bactrocera cucurbitae	cuc	JN635562	-
Bactrocera diaphora	dia	NC_028347	*Zhang et al. 2015*
Bactrocera scutellata	scu	KT159731	-
Bactrocera tau	tau	NC_027290	*Tan et al. 2015*
Dacus longicornis	lon	KX345846	*Jiang et al. 2016*
Drosophila melanogaster	-	AF200828	-

Supplementary Table S2. List of the primers used for the amplification of the *Ceratitis fascivenṭris* mtDNA. The primer sequence, the size of the amplicon and the annealing temperature used at PCR reactions are given. Cc: primers based on the mtDNA sequence of *C. capitata*, Cf: primers based on the mtDNA sequence of *C. fasciventris*, Bd: primers based on the mtDNA sequence of *B. dorsalis.* Numbers in primer names indicate primer position on the respective mtDNA. GenBank accession numbers of the mtDNA sequences used are given in Supplementary Table S1. F: forward primer, R: reverse primer

Primer name	Sequence	Amplicon size	Annealing T
**Cc 354F**	5′-CAATCTCTTCTAATTCCTGATTAGG-3′	702 bp	53°C
**Cc1056R**	5′-AATCCAATAAATGGGGGTAATCC-3′		
**Cf819F**	5′-TTCTCTTCTATCAATCATTTAGG-3′	843 bp	50°C
**Cf1662R**	5′-TTAGTGCTCCTGGGTGAC-3′		
**Cc1508F**	5′-CTAAACTTCAGCCATTTAATCGC-3′	706 bp	56°C
**Cc2214R**	5′-GGTATAAAATAGGGTCTCCTCC-3′		
**Cc2020F**	5′-CAGTAATTAATATACGATCTACCG-3′	829 bp	54°C
**Cc2849R**	5′-ATAGTCTGAATATCGCCGAGG-3′		
**Cc2660F**	5′-TTTATCAATAGGAGCAGTATTTGC-3′	727 bp	54°C
**Cc3387R**	5′-ATAACCGAAGAGAAGGAAAAGC-3′		
**Cf3162F**	5′-CTCCAAGATAGAGCTTCACC-3′	920 bp	51°C
**Cf4082R**	5′-TAGGGGATTGAGGAATTACTG-3′		
**Cc3826F**	5′-TTTTAATCATTAGATGACTGAAAGC-3′	825 bp	54°C
**Cc4651R**	5′-AAGTAAGAAGAAGATGTCCTGC-3′		
**Cc4451F**	5′-TACCTCTTTGATTATGTTTTATGC-3′	596 bp	53°C
**Cc5047R**	5′-CGTAAACCTAATGTGACTGG-3′		
**Cf4876F**	5′-GTGCTATAACATCCGTTTCAGG-3′	944 bp	52°C
**Cf5820R**	5′-GATCAAAACCGCATTCAAATGG-3′		
**Bd5623F**	5′-ATTTGACTTCCAATCATAAGGTC -3′	817 bp	53°C
**Bd6439R**	5′-GCTTAAAATAGAGCATAACACTG-3′		
**Cf6260F**	5′-TTTATCTTTTAATGGTTAAACTCC-3′	755 bp	50°C
**Cf7015R**	5′-TAACGGTTTGTTATTCTTTTCG-3′		
**Cc6909F**	5′-TCCCCGTAATATAATTCAACCC-3′	827 bp	54°C
**Cc7736R**	5′-GGAGATGTCGCTTTTTTATTAGC-3′		
**Cc7520F**	5′-AATGAACCAAAGCAGAAACTGG-3′	843 bp	55°C
**Cc8363R**	5′-CATTTAGTGGGGGAAATATTCG-3′		
**Cf8197F**	5′-GAATAATTCATATCATTGACACCAC-3′	934 bp	52°C
**Cc9131R**	5′-TTAGTTTCTTTACCTTTATTAGTAGG-3′		
**Cc8948F**	5′-TTATTGACCCAGATACAGGAGC-3′	743 bp	53°C
**Cc9691R**	5′-GAGTATGTGAAGGTGCTTTAGG-3′		
**Cc9543F**	5′-AACCTTGTATAAAACAAATAGGAC-3′	708 bp	53°C
**Cc10251R**	5′-CATTAGATGCCAAAGATGTAAC-3′		
**Cf10075F**	5′-TTATTCAAATAAATCATCCTCTAGC-3′	733 bp	52°C
**Cf10808R**	5′-CCGTTAGCGTGTATAGTTCG-3′		
**Cc10702F**	5′-CCTTGCTATACATTACACAGC-3′	725 bp	54°C
**Cc11427R**	5′-ATTACTCCTCCTAGTTTATTAGG-3′		
**Cf11289F**	5′-ATCCTTATTTACTAGGTGATCCAG-3′	801 bp	53 °C
**Cf12090R**	5′-AAGGTGAATCTGAATTAGTATCAGG3′		
**Cc11861F**	5′-TAACGAAAACGAGGTAAAGTCCC-3′	760 bp	55°C
**Bd12620R**	5′-TTTTGGAACGGAAGGTTCTAGG-3′		
**Bd12529F**	5′-CGCATCACAAAAAGGTTGAGG-3′	684 bp	55°C
**Bd13213R**	5′-AGACGAGAAGACCCTATAAATC-3′		
**Bd13004F**	5′-TCTCCAAAAAAATTACGCTGTTATCCC-3′	926 bp	55°C
**Bd13929R**	5′-ATTGTACCTTGTGTATCAGGGTTTATC-3′		
**Cf13500F**	5′-ATACTTTAACATTACAAAACTTTAGAC-3′	898 bp	50 °C
**Cf14399R**	5′-GCTTATGTTTAAGAAGAGATGG-3′		
**Cc14238F**	5′-ACCTTAATAGTAAGAGTGACGG-3′	576 bp	54°C
**Cc14814R**	5′-GTTAAATTTGTGCCAGCATCC-3′		
**Cf14576F**	5′-GTTTCAAGAACATAACTAATACTACC-3′	1082 bp	49°C
**Cf15658R**	5′-CAAATTGTTATTTATTTCAGAATTTTAGG-3′		
**Cc15083F**	5′-AAAATTTAGGTATCTCCTTCCC-3′	715 bp	56°C
**Cc15798R**	5′-AATGCCAATATAGACATTTCCG-3′		
**Cc15773F**	5′-CTCTCGGAAATGTCTATATTGG-3′	753 bp	56°C
**Cc509R**	5′-CAAACAATAAAACAGCAGATGC-3′		

Supplementary Figure S1. Comparison of the 2L polytene chromosome arms of *Ceratitis fasciventris* and *Ceratitis capitata*. (a) The 2L polytene chromosome arm of *C. fasciventris*; (b) The 2L polytene chromosome arm of *C. capitata*. Dot lines connect characteristic landmarks of the two chromosomes. C indicates the centromere. Note the overall banding pattern similarity.

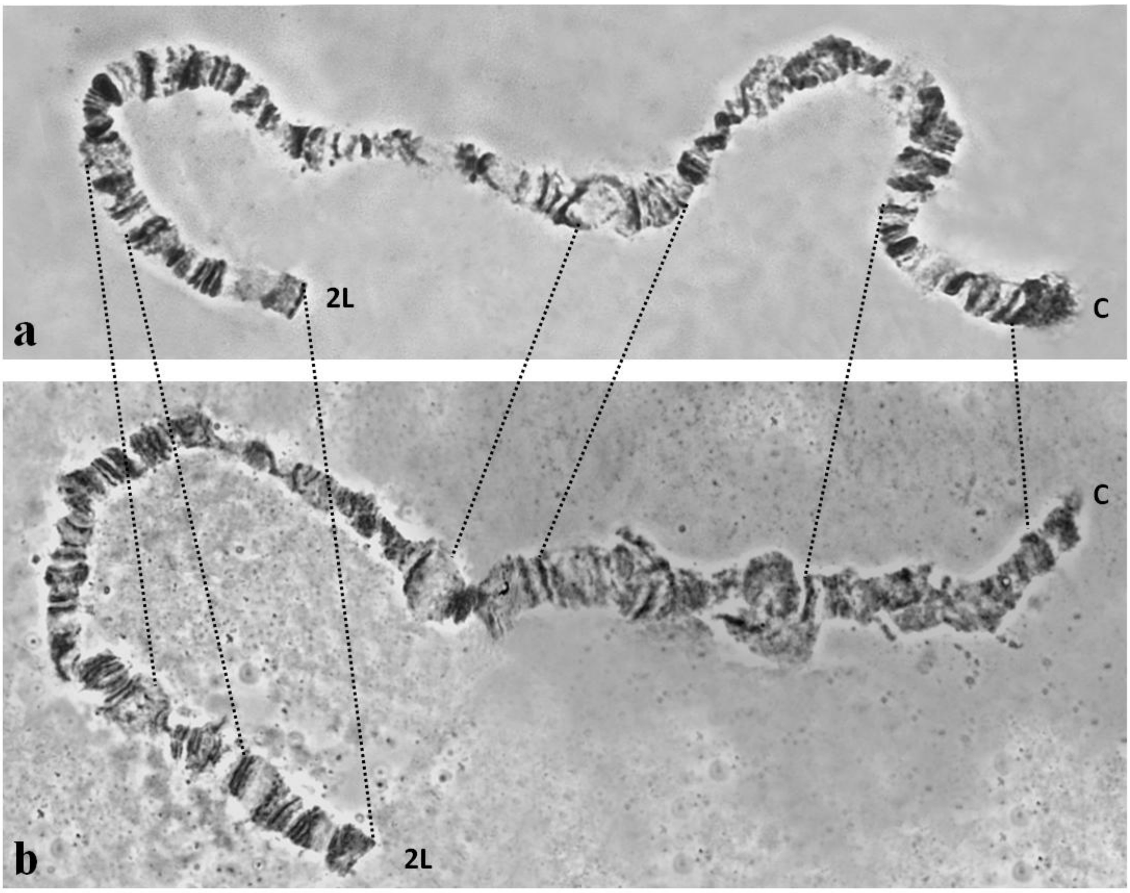

Supplementary Figure S2. Comparison of the 2R polytene chromosome arms of *Ceratitis fasciventris* and *Ceratitis capitata*. (a) The 2R polytene chromosome arm of *C. fasciventris*; (b) The 2R polytene chromosome arm of *C. capitata*. Dot lines connect characteristic landmarks of the two chromosomes. C indicates the centromere. Note the overall banding pattern similarity.

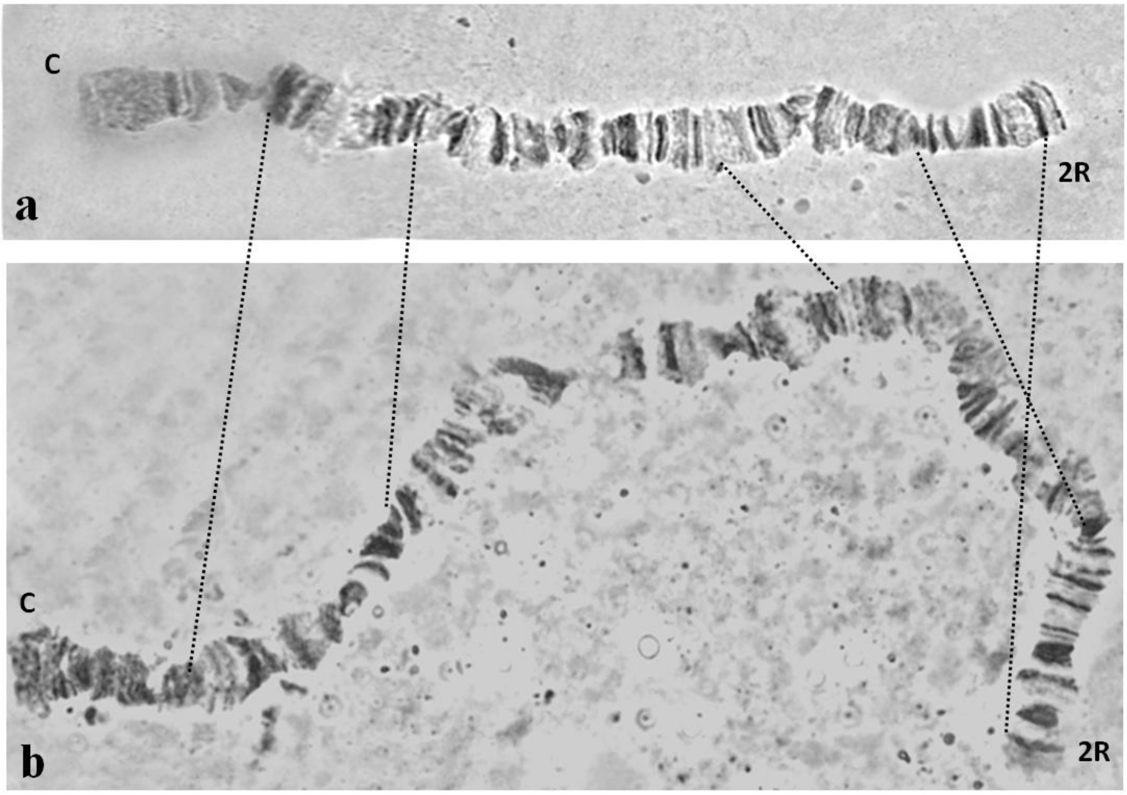

Supplementary Figure S3. Comparison of the 3R polytene chromosome arms of *Ceratitis fasciventris* and *Ceratitis capitata*. (a) The 3R polytene chromosome arm of *C. fasciventris*; (b) The 3R polytene chromosome arm of *C. capitata*. Dot lines connect characteristic landmarks of the two chromosomes. C indicates the centromere. Note the overall banding pattern similarity.

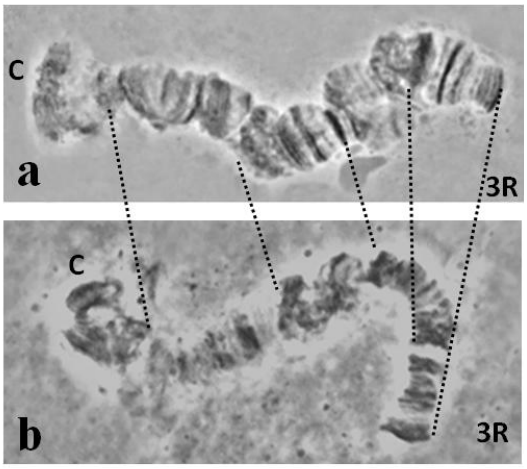

Supplementary Figure S4. Comparison of the 4L polytene chromosome arms of *Ceratitis fasciventris* and *Ceratitis capitata*. (a) The 4L polytene chromosome arm of *C. fasciventris*; (b) The 4L polytene chromosome arm of *C. capitata*. Dot lines connect characteristic landmarks of the two chromosomes. C indicates the centromere. Note the overall banding pattern similarity.

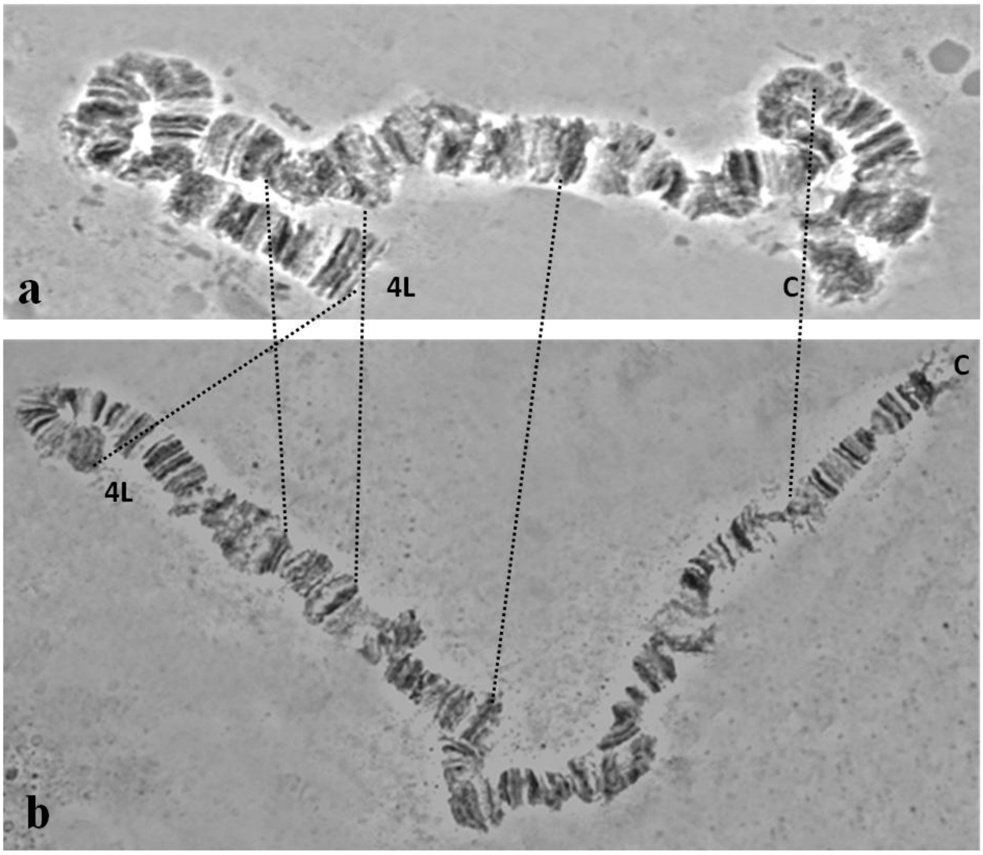

Supplementary Figure S5. Comparison of the 4R polytene chromosome arms of *Ceratitis fasciventris* and *Ceratitis capitata*. (a) The 4R polytene chromosome arm of *C. fasciventris*; (b) The 4R polytene chromosome arm of *C. capitata*. Dot lines connect characteristic landmarks of the two chromosomes. C indicates the centromere. Note the overall banding pattern similarity.

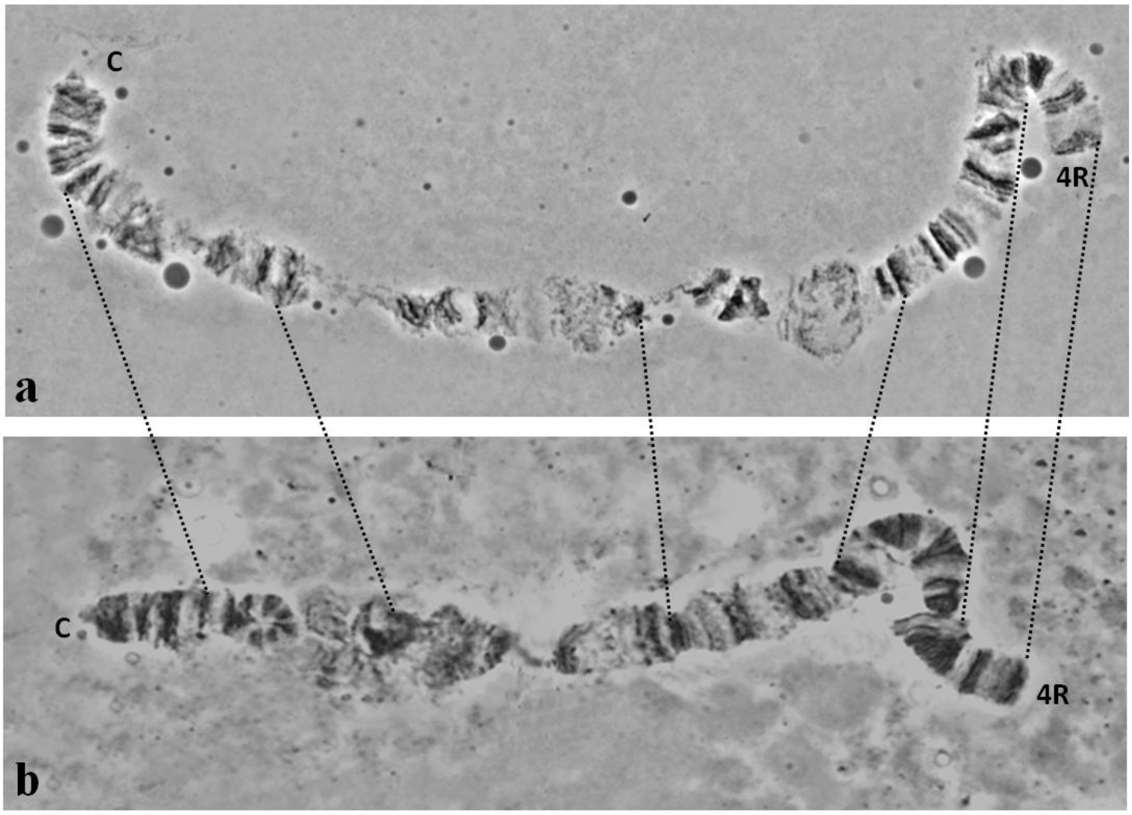

Supplementary Figure S6. Comparison of the 5R polytene chromosome arms of *Ceratitis fasciventris* and *Ceratitis capitata*. (a) The 5R polytene chromosome arm of *C. fasciventris*; (b) The 5R polytene chromosome arm of *C. capitata*. Dot lines connect characteristic landmarks of the two chromosomes. C indicates the centromere. Note the overall banding pattern similarity.

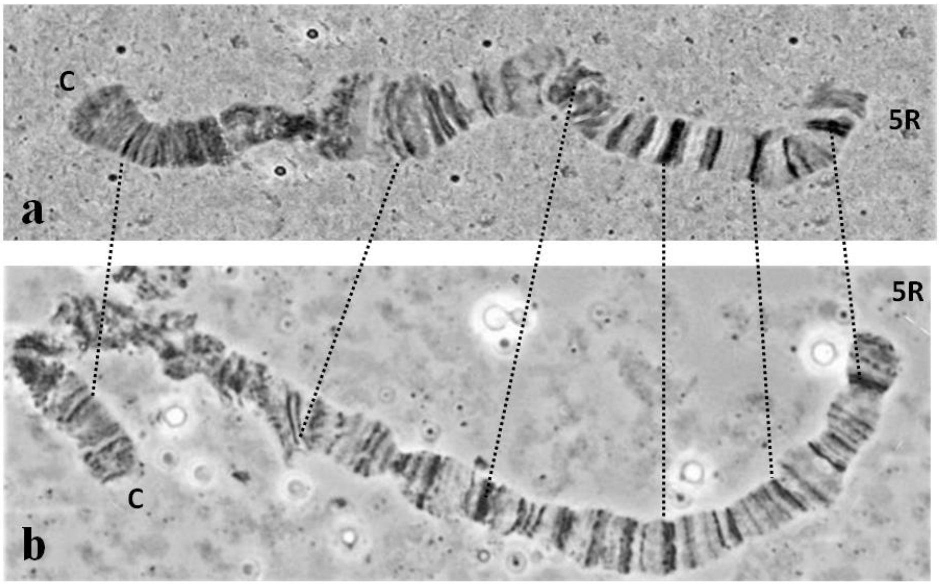

Supplementary Figure S7. Comparison of the 6L polytene chromosome arms of *Ceratitis fasciventris* and *Ceratitis capitata*. (a) The 6L polytene chromosome arm of *C. fasciventris*; (b) The 6L polytene chromosome arm of *C. capitata*. Dot lines connect characteristic landmarks of the two chromosomes. C indicates the centromere. Note the overall banding pattern similarity.

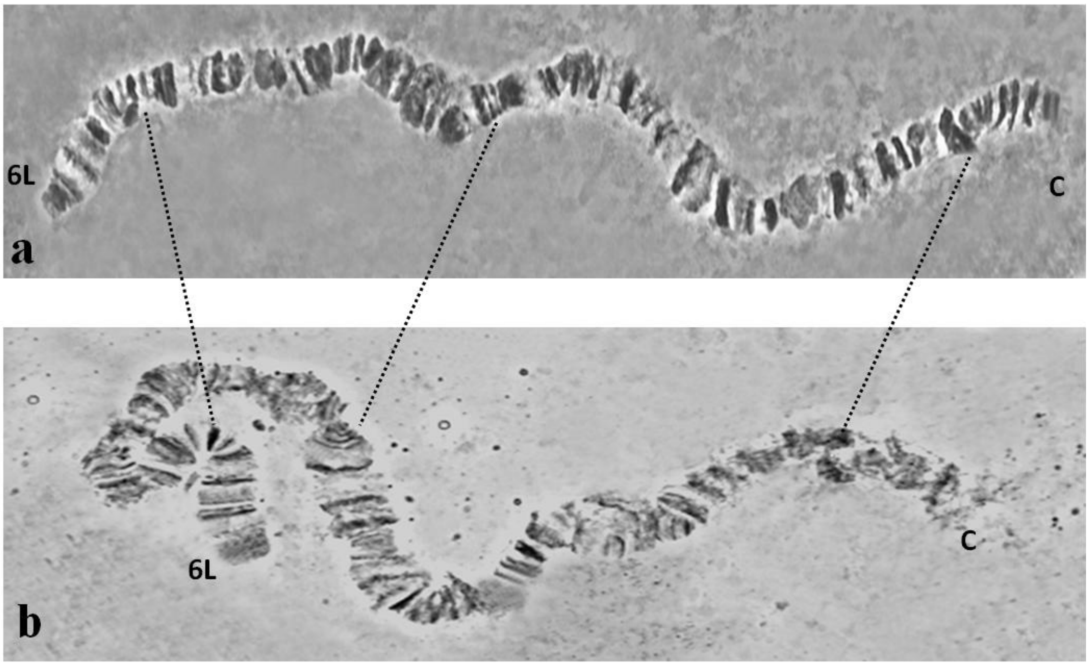

Supplementary Figure S8. Comparison of the 6R polytene chromosome arms of *Ceratitis fasciventris* and *Ceratitis capitata*. (a) The 6R polytene chromosome arm of *C. fasciventris*; (b) The 6R polytene chromosome arm of *C. capitata*. Dot lines connect characteristic landmarks of the two chromosomes. C indicates the centromere. Note the overall banding pattern similarity.

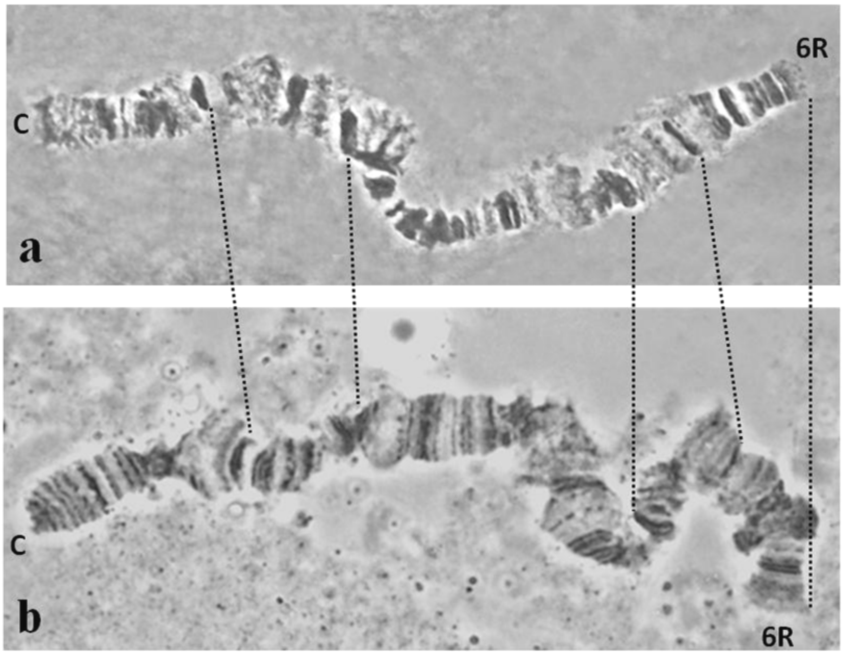

Supplement: Supplementary file 1 — Supplementary Information [file 41598_2017_5132_MOESM1_ESM.pdf]
